# Negative pressure pulmonary edema in a patient undergoing open rhinoplasty: A case report

**DOI:** 10.1097/MD.0000000000024240

**Published:** 2021-01-08

**Authors:** Hanwool Park, Sugeun Nam, Yong Ju Jang, Seungwoo Ku, Seong-Soo Choi

**Affiliations:** a Department of Anesthesiology and Pain Medicine; b Department of Otorhinolaryngology, Asan Medical Center, University of Ulsan College of Medicine, Seoul, Korea.

**Keywords:** nasal packing, negative pressure pulmonary edema, open rhinoplasty

## Abstract

**Rationale:**

Negative pressure pulmonary edema (NPPE) is associated with serious postoperative complications. Compact nasal packing is always done after an open rhinoplasty procedure which makes it difficult to achieve positive pressure ventilation via a mask if NPPE arises.

**Patient concerns:**

A 21-year-old healthy man got an open rhinoplasty, septal perforation repair, and revisional septal reconstruction. After surgery, he became so agitated that it was difficult to calm him. We decided to remove the endotracheal tube. On arrival at the post-anesthesia care unit, he was cyanotic and his SpO_2_ had decreased to about 2%. We attempted positive pressure ventilation using mask bagging; however, it was ineffective due to the nasal packing.

**Diagnoses:**

Negative pressure pulmonary edema

**Interventions:**

Emergent reintubation was immediately done and Ambu bagging was commenced. A considerable pinkish secretion came out of the tube. A T-piece was applied to him using 15 L/min of oxygen supply. The patient was eventually transferred to the intensive care unit of our hospital.

**Outcomes:**

On postoperative day (POD) 1, a decision was made to extubate, and the oxygen supply was shifted to 3L/min using a venturi-mask. On POD 2, a chest posteroanterior radiograph was taken and indicated no active lung lesion. The patient was subsequently discharged without any complications. He had no symptoms on POD 6, 11, and 18 at follow-up visits to our outpatient clinic.

**Lessons:**

Anesthesiologists should be alert to the possibility of NPPE and its treatment because of its rapid onset but positive clinical outcome if there is a proper intervention. In nasal surgery cases in particular, early re-intubation should be conducted and extubation should be done to fully awaken the patients.

## 1 Introduction

Negative pressure pulmonary edema (NPPE) is rare but can have serious consequences for the patient due to the resulting severe hypoxia.^[1]^ NPPE is caused by forceful inspiration against the closed upper airway. This causes significant negative pressure on the lung and results in pulmonary edema.^[1]^ Laryngospasm is the most common cause of NPPE but other upper airway obstructions (eg, choking) can also lead to this condition.^[2]^ Early detection and prompt management are necessary to reduce the morbidity and mortality associated with NPPE and effective airway management and immediate ventilation are an essential part of this intervention. It is also known that upper respiratory tract surgery is a risk factor for laryngospasm.^[3]^ Hence, some anesthesiologists recommend spraying the larynx with topical anesthesia before intubation during rhinoplasty surgery.^[3]^ Of note also, nasal packing is typically mandatory after open rhinoplasty to compress the wound and maintain the shape of the corrected nose. A complication of this however is that the patient can have subsequent difficulty in breathing. Furthermore, mask ventilation may be difficult if positive pressure ventilation is required in this situation. Quick and accurate decision-making for airway management including endotracheal intubation is therefore key when NPPE arises in rhinoplasty patients. In our current case report, we describe a case of NPPE in a 21-year-old healthy man who had undergone open rhinoplasty. We discuss the airway management of NPPE in open rhinoplasty.

## 2 Case report

A 21-year-old healthy man (height 174 cm, weight 71 kg, body mass index 23.45 kg/m^2^) was scheduled for an open rhinoplasty, septal perforation repair, and revisional septal reconstruction at our hospital. He had previously been healthy. The results of a chest posteroanterior (PA) radiogram, electrocardiogram and other vital signs (blood pressure, heart rate, respiratory rate, body temperature) were normal. At the time of the surgery, he was anesthetized using sevoflurane (volatile induction and maintenance). After checking his airway, rocuronium (40 mg) was injected intravenously. Intubation was done using an RAE tube (7.5 mm internal diameter, 23 cm oral depth) without any trauma and the auscultation sound was normal in both lungs. The anesthesia was maintained using sevoflurane (2 vol%) and N_2_O (50%). There were no abnormal events during the 3-hour rhinoplasty operation in which 300 mL of crystalloid was injected. At the end of the surgery, pyridostigmine (15 mg) and glycopyrrolate (0.4 mg) were injected to reverse the residual muscle relaxation.

**Figure 1:**
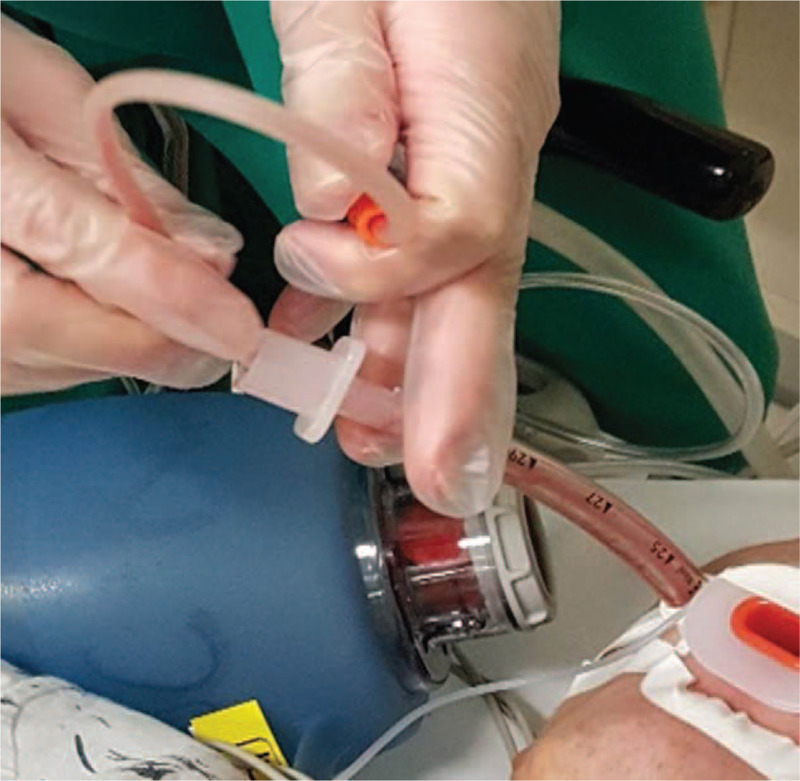
Pinkish secretion emerging from the endotracheal tube in the present study case.

**Figure 2:**
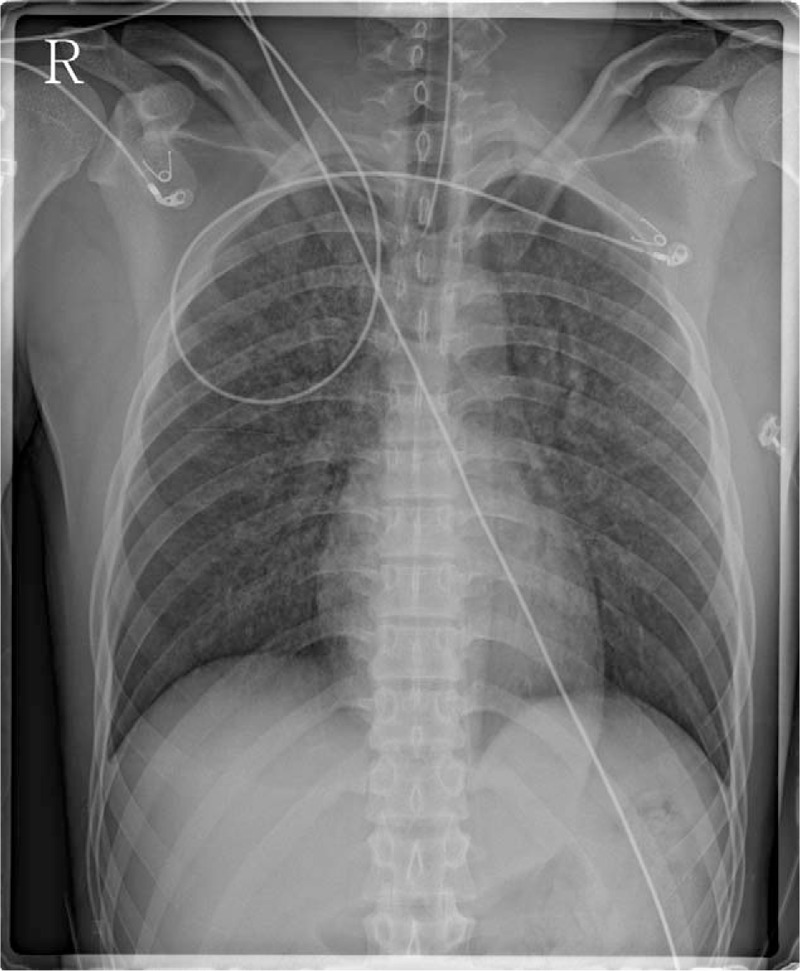
Portable chest anteroposterior radiograph obtained 17 minutes after re-intubation showing diffuse alveolar infiltration in both lungs.

The patient's nose was totally occluded following the nasal packing. When he was regained consciousness, however, he became so agitated that it was difficult to calm him. He elevated his head over 5 seconds and it was thus decided to remove the endotracheal tube. He then became calmer and was breathing well. After confirming an SpO_2_ level of 100%, the patient was placed in the post-anesthesia care unit (PACU) within 3 minutes. On arrival at the PACU, however, he was cyanotic and his SpO_2_ had decreased to about 2%. He was also drowsy and noncooperative. An arterial pulse was prominent, and the initial blood pressure was 134/74 mmHg. We determined that he was in respiratory arrest and attempted positive pressure ventilation using mask bagging. This was ineffective however due to the nasal packing. Emergent reintubation was immediately done and Ambu bagging was commenced. Simultaneously, an arterial line was inserted and subsequent arterial blood gas analysis (ABGA) indicated a pH of 7.24, PaCO_2_ of 55 mmHg, pO_2_ of 61 mmHg, and an SaO_2_ level at 86%. At 5 minutes from the re-intubation, suctioning of the endotracheal tube was commenced and a considerable pinkish secretion came out of the tube (Fig. 1). At 17 minutes from the re-intubation, a portable chest anteroposterior radiograph was taken and revealed peribronchial infiltration in both lung fields (Fig. 2). At 27 minutes after re-intubation, the patient was alert and ventilating by himself. The ABGA results at this stage were a pH of 7.45, pCO_2_ of 32 mmHg, pO_2_ of 94 mmHg, and SpO_2_ level of 98%. A T-piece was applied to him using 15 L/min of oxygen supply.

**Figure 3:**
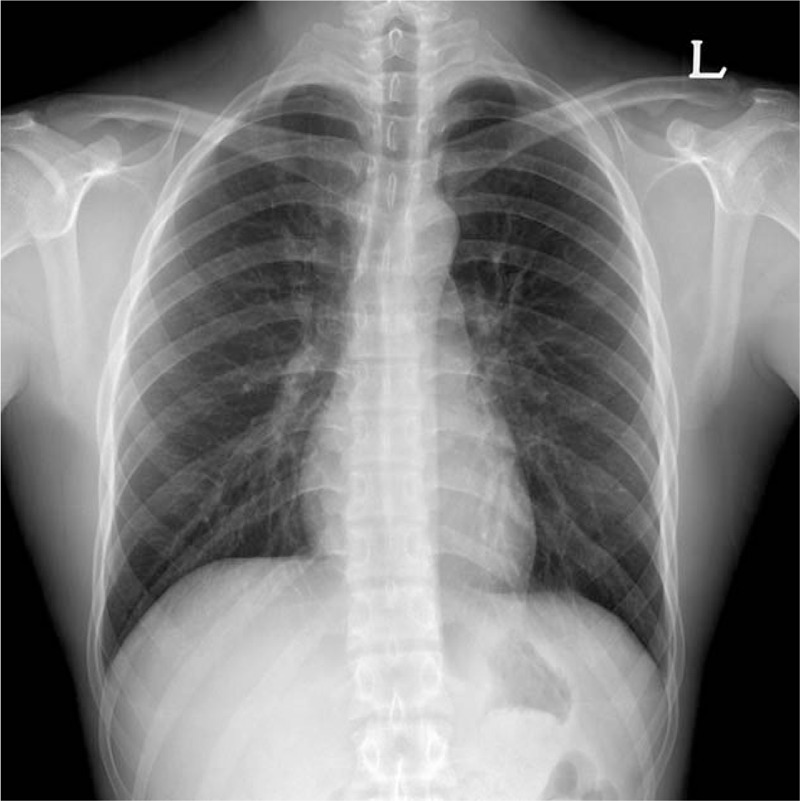
Chest posteroanterior radiogram at POD2 indicating no active lung lesion.

The patient was eventually transferred to the intensive care unit of our hospital and his oxygen supply was set to 9L/min. On postoperative day (POD) 1, the ABGA measurements included a pH of 7.43, PaCO_2_ of 38 mmHg, pO_2_ of 196.5 mmHg, and an SpO_2_ of 100%. A decision was then made to extubate, and the oxygen supply was shifted to 3 L/min using a venturi-mask. At 5 hours from the extubation, the ABGA readings were as follows:

pH, 7.43; pCO_2,_ 39 mmHg; pO_2_, 80 mmHg, and SpO_2_ 96%. The patient was then transferred to the general ward. On POD 2, a chest PA radiograph was taken and indicated no active lung lesion (Fig. 3). The patient was subsequently discharged without any complications. He had no symptoms on POD 6, 11, and 18 at follow-up visits to our outpatient clinic.

## 3 Discussion

NPPE is a type of noncardiogenic pulmonary edema that is mainly caused by a high negative intrathoracic pressure that has built up due to an upper airway obstruction.^[4]^ NPPE cases began to be described, and the mechanisms analyzed, in the 1970s.^[5]^ The reported incidence of NPPE among laryngospasm cases is 0.1%.^[6]^

NPPE is classified as type I or type II.^[1]^ Type I NPPE is caused by a forceful inspiratory effort in cases of acute airway obstruction such as post-extubation laryngospasm, epiglottitis, choking, or hanging. Type II NPPE is caused by relief of a chronic partial airway obstruction such as post-tonsillectomy, post-removal of an upper airway tumor, choanal stenosis, or hypertrophic redundant uvula. The pathophysiology of type I NPPE involves fluid shifts due to swings in the intrathoracic pressure. During the inspiration against an occluded airway, the intrathoracic pressure drops to −140 cmH_2_O. This produces an increased venous return and afterload. The resulting pulmonary blood volume and pulmonary venous pressure increase generates a higher hydrostatic pressure and causes pulmonary edema. This pathophysiology explains why a healthy individual can have an increased incidence of NPPE.^[7]^ The pathophysiology of type II NPPE is less clear than that of type I. In these cases, a chronic obstructive airway produces positive pleural and alveolar pressure, and results in a decreased venous return and pulmonary blood volume. Sudden relief of an upper airway obstruction induces an increased venous return and preload. This causes a hydrostatic pressure elevation and pulmonary edema.

An airway obstruction leading to NPPE in adults is most often reported in the context of post-extubation laryngospasm following surgery.^[7]^ The symptoms of NPPE include tachypnea, tachycardia, rales, decreased SpO_2_, and a pinkish sputum. Chest x-rays in affected patients indicate rapid bilateral changes consistent with pulmonary edema. Early detection and maintenance of a patent airway are critical for treating NPPE. Most cases of NPPE resolve within 24 to 48 hours when treated properly.^[1,6,7]^ Endotracheal intubation and positive-pressure ventilation with supplemental oxygen are an essential part of this treatment. In some cases, however, only continuous positive airway pressure via facial mask treatment can resolve this condition.^[6]^

In open rhinoplasty surgeries, bilateral nasal packing is used to prevent adhesions between the septum and lateral wall structures and reduce postoperative bleeding. This usually results in only minor complications such as a slight increase in the pCO_2_ and marginal pO_2_ reduction.^[8]^ However, this can be very harmful in NPPE cases because of the difficulties in breathing and airway obstruction. Several NPPE cases have reported following nasal surgery.^[9–11]^ In 2006, Westreich et al^[3]^ reviewed 146 NPPE patients of which 8% had undergone intranasal surgery. The nasal packing requirements of these procedures reduce the efficiency of mask ventilation after extubation. This presents a potentially very serious clinical issue if NPPE occurs and re-intubation is essential.

A fully alert patient before extubation is important for the prevention of NPPE because a residual muscle blockade can be a risk factor for this condition.^[12]^ In our current case, the patient had become so distressed and difficult to calm that that we had to extubate him far earlier than normal. Agitation such as this often leads to a hyperadrenergic state and systemic hypertension, serving to further increase the ventricular afterload and an elevation of the pulmonary pressure.^[7]^

In conclusion, anesthesiologists should be alert to the possibility of NPPE and its treatment because of its rapid onset but positive clinical outcome if there is a proper intervention. In nasal surgery cases in particular, early re-intubation should be conducted and extubation should be done to fully awaken the patients.

## Author contributions

**Data curation:** Hanwool Park, Sugeun Nam, Seungwoo Ku, Seong-Soo Choi

**Informed consent:** we got an informed consent from the patient for the publication of this report.

**Writing – original draft:** Hanwool Park, Seong-Soo Choi.

**Writing – review & editing:** Yong Ju Jang, Seungwoo Ku, Seong-Soo Choi.
